# Omeprazole and risk of osteoarthritis: insights from a mendelian randomization study in the UK Biobank

**DOI:** 10.1186/s12967-024-05255-y

**Published:** 2024-05-27

**Authors:** Siyang Cao, Yihao Wei, Yaohang Yue, Guoqing Li, Hongli Wang, Jianjing Lin, Qichang Wang, Peng Liu, Fei Yu, Ao Xiong, Hui Zeng

**Affiliations:** 1 National & Local Joint Engineering Research Centre of Orthopaedic Biomaterials, Peking University Shenzhen Hospital, Shenzhen, Guangdong People’s Republic of China; 2 Shenzhen Key Laboratory of Orthopaedic Diseases and Biomaterials Research, Peking University Shenzhen Hospital, Shenzhen, Guangdong People’s Republic of China; 3 Department of Bone & Joint Surgery, Peking University Shenzhen Hospital, Shenzhen, Guangdong People’s Republic of China; 4 Department of Orthopaedics, Beijing Jishuitan Hospital, Capital Medical University, Beijing, People’s Republic of China; 5 Department of Rheumatism and Immunology, Peking University Shenzhen Hospital, Shenzhen, Guangdong People’s Republic of China; 6 Department of Sports Medicine and Rehabilitation, Peking University Shenzhen Hospital, Shenzhen, Guangdong People’s Republic of China

**Keywords:** Mendelian randomization, Osteoarthritis, Omeprazole, Causal association

## Abstract

**Background:**

A former cohort study has raised concern regarding the unanticipated hazard of omeprazole in expediting osteoarthritis (OA) advancement. The precise nature of their causal evidence, however, remains undetermined. The present research endeavors to investigate the underlying causal link between omeprazole and OA through the application of mendelian randomization (MR) analysis.

**Methods:**

The study incorporated the ukb-a-106 and ukb-b-14,486 datasets. The investigation of causal effects employed methodologies such as MR-Egger, Weighted median, Inverse variance weighted (IVW) with multiplicative random effects, and IVW (fixed effects). The IVW approach was predominantly considered for result interpretation. Sensitivity analysis was conducted, encompassing assessments for heterogeneity, horizontal pleiotropy, and the Leave-one-out techniques.

**Results:**

The outcomes of the MR analysis indicated a causal relationship between omeprazole and OA, with omeprazole identified as a contributing risk factor for OA development (IVW model: *OR* = 1.2473, *P* < 0.01 in ukb-a-106; *OR* = 1.1288, *P* < 0.05 in ukb-b-14,486). The sensitivity analysis underscored the robustness and dependability of the above-mentioned analytical findings.

**Conclusion:**

This study, employing MR, reveals that omeprazole, as an exposure factor, elevates the risk of OA. Considering the drug’s efficacy and associated adverse events, clinical practitioners should exercise caution regarding prolonged omeprazole use, particularly in populations with heightened OA risks. Further robust and high-quality research is warranted to validate our findings and guide clinical practice.

## Graphical Abstract

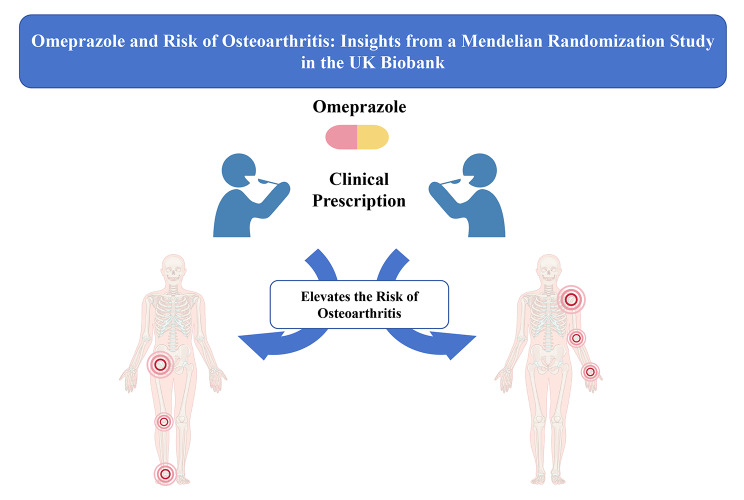

## Introduction

Osteoarthritis (OA), the most prevalent form of arthritis [1], affects around 7% of the global population [2]. With the aging population and increasing obesity rates, the incidence of OA is projected to rise [3], imposing a significant socioeconomic burden on nations worldwide [4]. To date, there has been no remedy capable of effectively halting the progression of OA. Clinical intervention primarily focuses on symptom management through approaches such as oral administration of non-steroidal anti-inflammatory drugs (NSAIDs) [5]. However, the use of NSAIDs is linked to a range of adverse effects [6, 7], such as gastrointestinal complications [7]. It is recommended to prescribe proton pump inhibitors (PPIs) along with NSAIDs as a cost-effective treatment for OA patients with moderate or high gastrointestinal risk [8–11]. As a result, PPIs are now one of the most frequently prescribed medications for OA patients.

While omeprazole, one of the most extensively prescribed PPIs, is generally considered safe and well-tolerated, concerns have arisen regarding its potential adverse effect of hypomagnesemia [12–14]. Magnesium, an essential ion, plays a crucial role in supporting and maintaining health [15]. Several observational studies in humans have demonstrated a negative correlation between either dietary magnesium intake or serum magnesium levels and the prevalence of radiographic knee OA [16–19]. Furthermore, a prospective cohort study found that decreased dietary magnesium intake was linked to poorer pain and function in individuals with radiographic knee OA [20]. Notably, a recent cohort study based on the general population has brought attention to the unexpected potential of omeprazole in accelerating the progression of OA [21]. Given confounding variables and the potential for reverse causation, the observational study can only establish an association between exposure and an event without being able to establish causation. At the pinnacle of the evidence hierarchy for studying therapeutic impacts on diseases stands the randomized controlled trial (RCT) [22]. Despite their effectiveness, RCTs can pose significant financial and temporal burdens, and in certain scenarios, the allocation of exposure may be deemed unethical or impractical [23–25].

Since the publication of a seminal article by Smith and Ebrahim at the beginning of the 21st century, mendelian randomization (MR) has garnered growing interest as a distinctive epidemiological method for inferring potential causal relationships using observational data [26]. It employs single nucleotide polymorphisms (SNPs) as instrumental variables for the exposure variable, enabling the estimation of a causal linkage between exposure and outcomes [27]. As the alleles of specific SNPs are randomly assigned at birth, genetic variations remain unaffected by potential confounders [28]. Furthermore, genetic variance is established prior to the disease’s onset, mitigating the possibility of reverse causation [29]. Evidence derived from genetically proxied exposure and disease outcome associations can be utilized to infer a causal link between exposure and outcome. Over the last decade, a plethora of MR studies have been undertaken to pinpoint causal factors linked to OA. For instance, MR investigations have offered backing for causal relationships between OA and elevated adiposity, coffee intake, bone mineral density, and sleep disruption, alongside diminished levels of serum calcium and low-density lipoprotein cholesterol [30]. These findings emphasize the potential advantages of decreasing weight and enhancing sleep quality to lower the risk of OA, while also highlighting the necessity for gaining a deeper understanding of the correlation between coffee intake and serum calcium concentrations regarding OA risk.

To address the identified research gap, this study aims to elucidate the causal relationship between omeprazole and OA through a two-sample MR analysis.

## Materials and methods

### Data sources

**Fig. 1:**
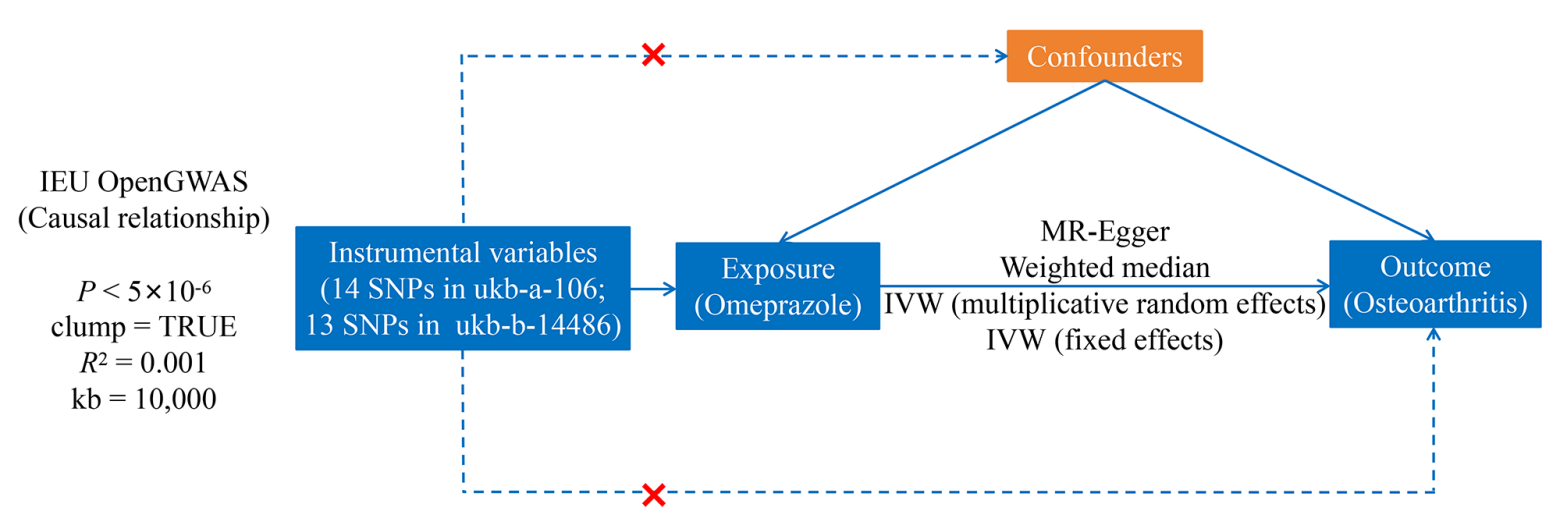
The overall design of the present Mendelian randomization analysis. **Abbreviations**: GWAS, Genome-Wide Association Study; IEU, integrative epidemiology unit; MR, mendelian randomization; SNP, single nucleotide polymorphism

The ukb-a-106 and ukb-b-14,486 datasets were acquired through querying the Integrative Epidemiology Unit Open Genome-Wide Association Study (GWAS) database (https://gwas.mrcieu.ac.uk/) using the search term “osteoarthritis”. The ukb-a-106 dataset incorporated SNPs data from 337,159 samples with OA (10,894,596 SNPs), while the ukb-b-14,486 dataset encompassed 9,851,867 SNPs sourced from a pool of 462,933 OA samples. As for GWAS data concerning omeprazole (ukb-a-129 dataset), it encompassed 337,159 samples and 10,894,596 SNPs. In this study, OA was considered the outcome variable, while omeprazole was examined as the exposure variable. Figure 1 provides a general overview of the present study design.

### Pre-processing of data

The MR studies are rooted in three essential principles: (1) a robust and statistically significant correlation between instrumental variables (IVs) and the focal exposure; (2) the IVs’ independence from potential confounders; (3) the exclusive effect of IVs on outcomes through the designated exposure, without alternative pathways. The exposure factors were assessed and IVs were screened using the “extract_instruments” feature within the TwoSampleMR package (*P* < 5 × 10^− 6^) [31]. Then, ‘clump’ was set to TRUE to remove the IVs for linkage disequilibrium analysis (*R*^2^ = 0.001 and kb = 10,000). An *R*^2^ value of 0 indicated complete linkage equilibrium between the two SNPs, meaning the assignment of these two SNPs was completely random. IVs displaying a significant correlation with the exposure factors were selected. Outcome variables were extracted using the ‘extract_outcome_data’ function from the R package. IVs from the exposure factors were merged with screening metrics, with the proxy setting set to TRUE. Furthermore, weak IVs were omitted based on the *F*-statistic for each SNP.

$$F=\frac{{R}^{2}}{1-{R}^{2}}\times \frac{N-K-1}{K}$$

Within this formula, *R*^*2*^ signified the cumulative explained variance attributed to the selected IVs concerning the exposure factors. *N* represented the sample size of the GWAS, while *K* indicated the number of screened SNPs for the exposure. An *F*-statistic of ≥ 10 indicated the absence of a weak instrumental bias, thereby highlighting the robust predictive capability of the IVs for the outcome.

### MR analysis

The ‘harmonize_data’ function within the TwoSampleMR package was utilized for the normalization of effect estimates. The key MR techniques included MR-Egger [32], Weighted median [33], and Inverse Variance Weighted (IVW), utilizing both multiplicative random effects and fixed effects [34]. Emphasis in the primary analysis was placed on the IVW method, which requires SNPs to fully comply with the three principles of MR research in order to obtain correct causal estimates. MR-Egger adds an additional intercept term, the main purpose of which is to determine the presence or absence of horizontal pleiotropy. The Weighted Median method assesses the presence or absence of causality using the majority of SNPs (majority of genetic variants). If there is no additional heterogeneity in the causal estimates for a given variable in IVW, then the results of the random effects and fixed effects will be the same, and therefore there is no loss of precision. In cases where the IVW method yields a noteworthy outcome (*P* < 0.05), it can be considered positive even if other methods’ results are not significant, given consistent directionality of *β*-values across methods and the absence of pleiotropy or heterogeneity. Subsequently, the findings were visually depicted using scatter plots, forest plots, and funnel plots. In the scatter plot, the IVW method remained the primary focus, with a very small intercept indicating that confounding factors had minimal influence and did not affect the reliability of the results. A positive slope of the line indicated a risk factor, while a negative slope indicated a protective factor. The forest plot was designed to assess the diagnostic efficacy of each SNP locus in predicting exposure factors for outcome diagnostics, with solid dots on the left indicating decreasing values and solid dots on the right indicating increasing values, focusing on the position of the IVW. Funnel plots were constructed to assess randomness, and if the IVs were symmetrically distributed along both sides of the IVW line, it indicated that MR conformed to Mendel’s second law of random grouping.

### Sensitivity analysis

To assess the robustness of the MR analysis outcomes, a sensitivity analysis was conducted. nitially, a heterogeneity test was conducted. A Cochran’s *Q* test resulting in a *P*-value above 0.05 indicated the lack of heterogeneity. Subsequently, a test for horizontal pleiotropy was conducted, and a *P*-value surpassing 0.05 suggested the absence of horizontal pleiotropic effects, indicating no confounding factors in the study. Finally, a Leave-One-Out (LOO) analysis was performed by iteratively removing each SNP. Consistency in the influence of the remaining SNPs on the outcome variable validated the reliability of the MR analysis results.

## Results

### Omeprazole is causally associated with OA

**Fig. 2:**
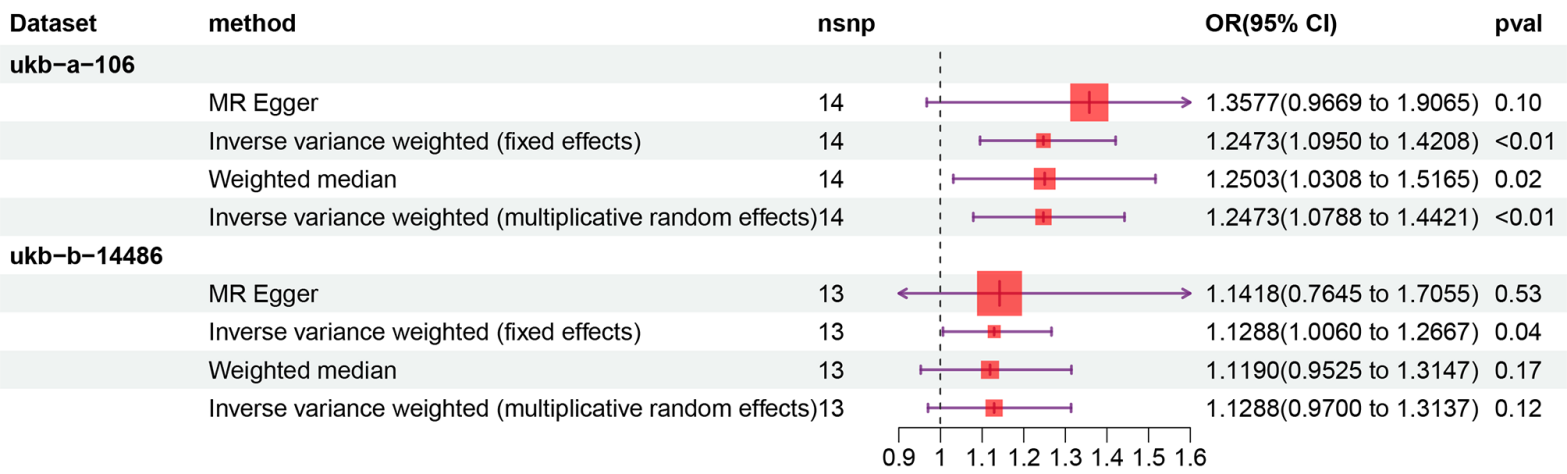
A forest plot illustrating the causal effect of omeprazole on the risk of osteoarthritis, generated using inverse variance weighting. **Abbreviations**: nsnp, number of single nucleotide polymorphisms; *OR*, odds ratio; *CI*, confidence interval, *p*val, *p*-value

**Fig. 3:**
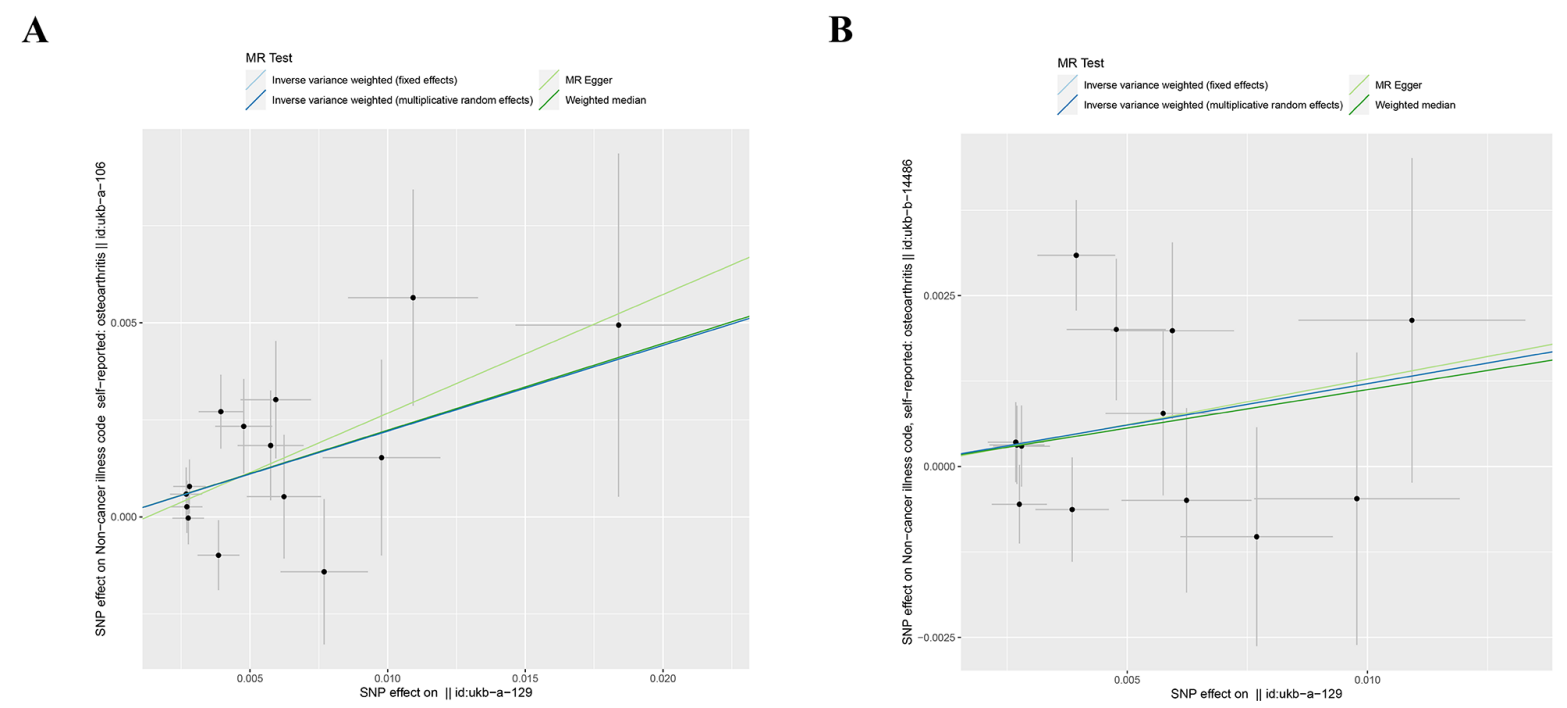
Scatter plots illustrating the causal relationships between omeprazole and osteoarthritis. **Abbreviations**: SNP, single nucleotide polymorphism

After careful screening, a total of 27 SNPs were identified as IVs, with 14 in the ukb-a-106 dataset and 13 in the ukb-b-14,486 dataset (Table 1). Subsequently, the impact of omeprazole on OA in the UK population was evaluated through MR analysis, with omeprazole as the exposure factor and OA as the outcome. MR outcomes from the four distinct methods consistently revealed a positive causal relationship between omeprazole and OA, indicating its role as a risk factor (IVW model: *OR* = 1.2473, *P* = 0.0009 in ukb-a-106; *OR* = 1.1288, *P* = 0.0392 in ukb-b-14,486) (Table 2; Fig. 2). To clarify the connection between exposure factors and outcomes, scatter plots of SNPs were generated. These scatter plots depicted a positive linear trend for omeprazole, indicating that heightened omeprazole expression corresponded to an increased likelihood of OA development (Fig. 3).

**Table 1:** Detailed genome-wide association study (GWAS) data on exposure factors and outcomes

	ukb-b-14,486	ukb-a-106	ukb-a-129
**Year**	2018	2017	2017
**Category**	Binary	N/A	N/A
**Sub category**	N/A	N/A	N/A
**Race**	European	European	European
**Gender**	Males and Females	Males and Females	Males and Females
**Number of Case Group**	38,472	28,257	19,668
**Number of Control Group**	424,461	308,902	317,491
**Sample Size**	462,933	337,159	337,159
**Number of SNPs**	9,851,867	10,894,596	10,894,596

**Abbreviations**: N/A, not applicable.

**Table 2:** Statistical data of mendelian randomization analysis

id.exposure	id.outcome	Method	nsnp	pval	OR	OR_lci95	OR_uci95
ukb-a-129	ukb-a-106	MR Egger	14	0.102845829	1.357714559	0.966916158	1.90646191
ukb-a-129	ukb-a-106	IVW (fixed effects)	14	0.000881874	1.247314763	1.094997458	1.420819843
ukb-a-129	ukb-a-106	Weighted median	14	0.023343524	1.250253647	1.030775067	1.516464874
ukb-a-129	ukb-a-106	IVW (multiplicative random effects)	14	0.002837256	1.247314763	1.078830588	1.442111611
ukb-a-129	ukb-b-14,486	MR Egger	13	0.530298196	1.141845427	0.764459198	1.705533772
ukb-a-129	ukb-b-14,486	IVW (fixed effects)	13	0.039234861	1.128829316	1.005996403	1.266660219
ukb-a-129	ukb-b-14,486	Weighted median	13	0.171416914	1.119005293	0.952468963	1.314659998
ukb-a-129	ukb-b-14,486	IVW (multiplicative random effects)	13	0.117376941	1.128829316	0.969959999	1.313719768

**Abbreviations**: IVW, Inverse variance weighted; lci95, lower 95% confidence interval; MR, mendelian randomization; nsnp, number of single nucleotide polymorphisms; *OR*, odds ratio; *p*val, p-value. uci95, upper 95% confidence interval

**Fig. 4:**
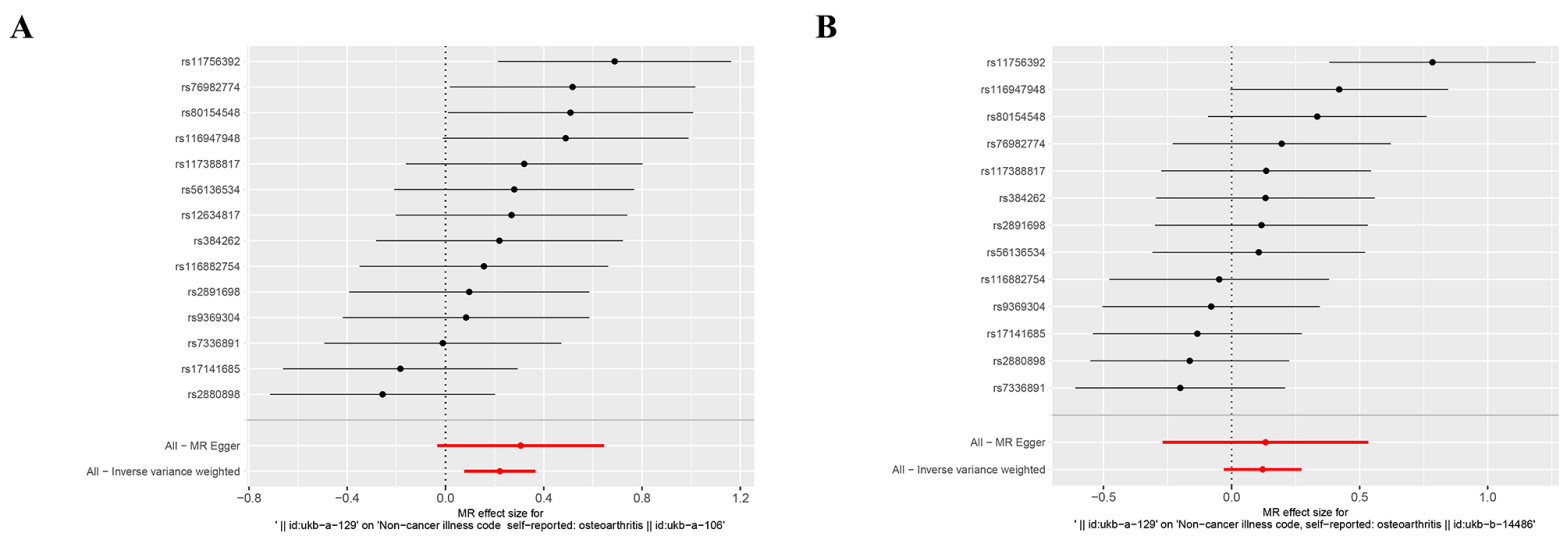
Single SNP effect combination forest plot of the causal relationships between omeprazole and osteoarthritis

**Fig. 5:**
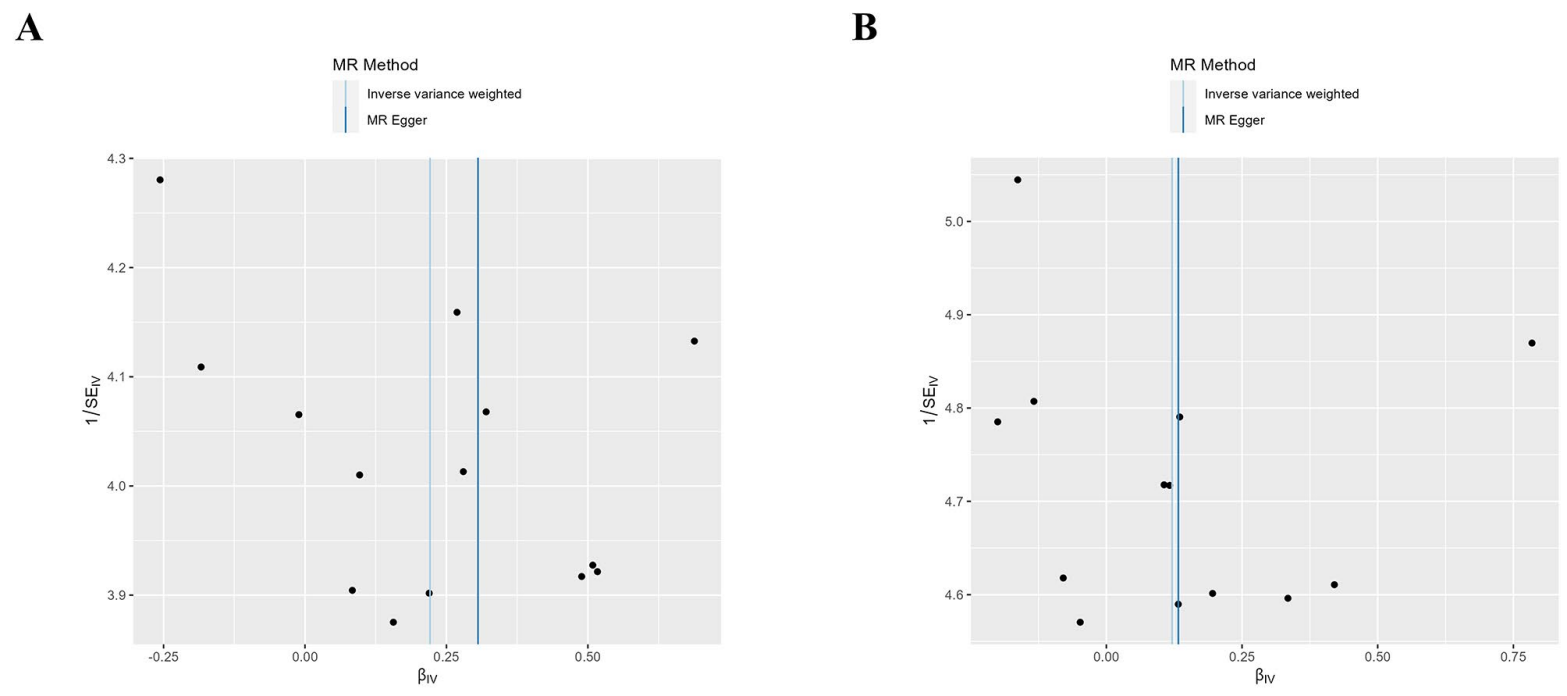
Funnel plots illustrating the causal relationships between omeprazole and osteoarthritis. **Abbreviations**: βiv, *β*-value of the instrumental variable

To assess the predictive efficacy of each SNP locus regarding exposure factors and outcomes, forest plots were constructed. In these plots, solid dots on the left represented lower risk, while solid dots on the right represented higher risk. The forest plot findings consistently positioned solid dots on the right side, indicating that elevated exposure factors increased the risk of disease onset according to the IVW approach (Fig. 4). Simultaneously, assessments of instrumental variable randomness were conducted and visualized through funnel plots. These plots showed a symmetrical distribution of IVs on both sides of the IVW line, confirming that the MR analysis adhered to the principles of MR grouping (Fig. 5).

### Sensitivity analysis

**Fig. 6:**
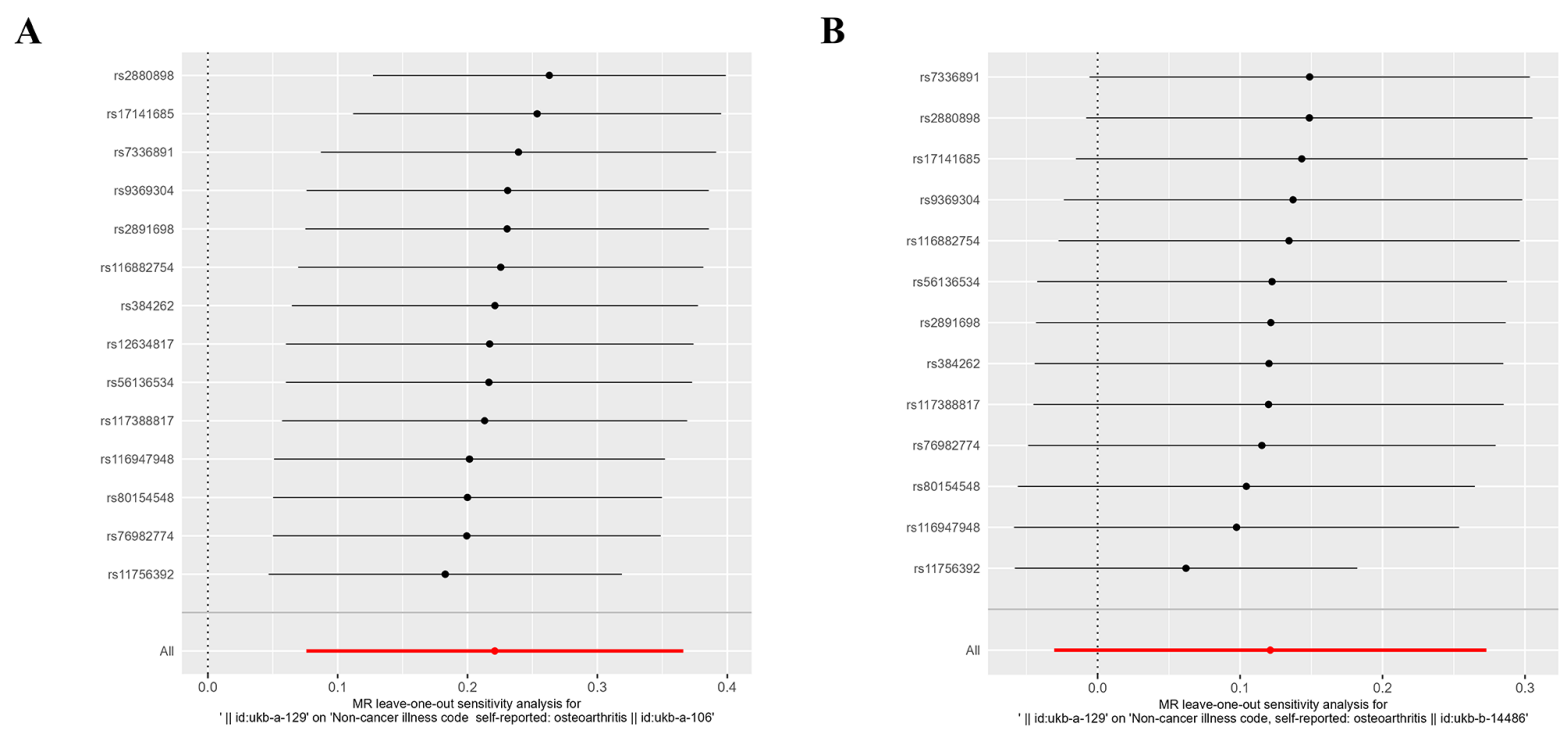
Forest plots depicting the leave-one-out analysis of the causal relationships between omeprazole and osteoarthritis

To ensure the robustness of the derived conclusions, a comprehensive sensitivity analysis was undertaken. First, all *Q*_pval values resulting from the heterogeneity tests exceeded 0.05, signifying the absence of heterogeneity among the UK samples and emphasizing the predominant employment of the IVW (fixed effects) method in the MR analysis (Table 3). Subsequently, MR-Egger and MR-PRESSO regression tests were implemented to monitor potential horizontal pleiotropy effects stemming from genetic IVs. The horizontal pleiotropy tests consistently revealed the absence of such effects involving omeprazole and OA (*P* > 0.05) (Tables 4 and 5). Furthermore, a LOO analysis was executed to assess the reliability of the outcomes. LOO, which involves the iterative exclusion of one SNP while utilizing the remainder for MR, gauges the extent to which a given SNP significantly influences the results. The primary objective of LOO is to ascertain whether the line connecting the black dots remains smooth, devoid of pronounced bias points. The outcomes of the LOO analysis demonstrated no significant bias points in the figures, thus affirming the reliability of the results (Fig. 6).

**Table 3:** Measures of heterogeneity using Cochran’s *Q* test

id.exposure	id.outcome	Exposure	Method	Q	Q_df	Q_pval
ukb-a-129	ukb-a-106	|| id: ukb-a-129	MR Egger	15.74883977	12	0.203012363
ukb-a-129	ukb-a-106	|| id: ukb-a-129	IVW	16.13893115	13	0.241708991
ukb-a-129	ukb-b-14,486	|| id: ukb-a-129	MR Egger	20.79579357	11	0.035549476
ukb-a-129	ukb-b-14,486	|| id: ukb-a-129	IVW	20.80281794	12	0.053343512

**Abbreviations**: IVW, Inverse variance weighted; MR, mendelian randomization; *Q*_df, degree of freedom associated with the Cochran Q test of heterogeneity; *Q*_pval, *p*-value of *Q*-test for heterogeneity test

**Table 4:** Examination of horizontal pleiotropy effects using MR-Egger regression tests

id.exposure	id.outcome	Exposure	egger_intercept	SE	pval
ukb-a-129	ukb-a-106	|| id: ukb-a-129	-0.000386716	0.00070932	0.595609568
ukb-a-129	ukb-b-14,486	|| id: ukb-a-129	-4.92E-05	0.000807106	0.952488135

**Abbreviations**: *p*val, *p*-value; SE, standard error

**Table 5:** Examination of horizontal pleiotropy effects using MR-PRESSO regression tests

	id.exposure	id.outcome	RSSobs	pval
**presso2**	ukb-a-129	ukb-a-106	18.84472855	0.26
**presso2.1**	ukb-a-129	ukb-b-14,486	24.58931261	0.077

**Abbreviations**: *p*val, *p*-value; RSSobs, observed residual sum of squares

## Discussion

With the aging population and dietary changes, the prevalence and incidence of OA are escalating annually [35]. Elderly patients suffering from OA may require the simultaneous or separate long-term use of medications like NSAIDs, corticosteroids, anticoagulants, and others. Clinicians frequently prescribe omeprazole as part of the treatment regimen for these patients, with the intent of providing gastric protection [36]. Recent evidence, however, suggests a potential unexpected risk of omeprazole related to accelerated OA progression [21].

The current investigation, utilizing publicly available GWAS data resources and employing a two-sample MR analysis, unravels, for the first time, a potential causal link between omeprazole and OA.

### Possible explanations

#### Hypomagnesemia

Magnesium ions, as a fundamental component within the human body, demonstrate a robust correlation with OA, impacting disease development and progression [16]. In a multicenter randomized controlled clinical trial involving 392 participants, researchers found that increased magnesium intake could potentially confer protective benefits against knee osteoarthritis (KOA) [37]. Conversely, a low intake of magnesium ions correlates with the gravity of OA, heightening the susceptibility to OA development and exacerbating knee joint discomfort [20, 37]. Magnesium insufficiency or diminished levels can induce an inflammatory state, thereby promoting the expression of cellular inflammatory factors such as tumor necrosis factor-α (TNF-α) and interleukin-6 (IL-6) [38, 39]. In animal experimentation, intra-articular injection of magnesium chloride to enhance magnesium ion levels acts as a mediator for hypoxia-inducible factor-1α, leading to reduced oxidative stress levels and effectively slowing the progression of KOA in rat models [40].

Omeprazole effectively halts gastric acid secretion by binding irreversibly to the hydrogen-potassium ATPase pump. Consequently, an increased pH within segments of the small intestine, induced by omeprazole, could potentially decrease the solubility of Mg2 + and hinder its absorption [14, 41]. Another presumed mechanism for the diminished absorption of magnesium by intestinal epithelial cells involves the PPIs-induced inhibition of transient receptor potential melastatin-6 (TRPM6) and TRPM7 channels [42]. A meta-analysis spanning nine observational studies, encompassing a total cohort of 109,798 patients, has unearthed a significant correlation between PPI use and the risk of hypomagnesemia [43]. Although the risk of hypomagnesemia is more pronounced in long-term PPIs users (typically exceeding one year), instances have also been documented within the first year of therapy initiation [44]. Given this potential risk, it is advisable to regularly assess serum magnesium levels in individuals on prolonged omeprazole regimens.

#### Vitamin B12 malabsorption

Significant causal relationships between serum vitamin B12 levels and musculoskeletal conditions, such as OA, have been observed [45]. Vitamin B12, by reducing blood homocysteine levels, has demonstrated potential protective effects against OA, particularly in weight-bearing joints and among females [46]. There are plausible mechanisms by which inhibiting gastric acid secretion may impair vitamin B12 absorption, ultimately resulting in deficiency. Parietal cells synthesize gastric acid and produce intrinsic factor, which is necessary for vitamin B12 absorption. Additionally, gastric acid is required to separate vitamin B12 from ingested dietary protein in order to facilitate its absorption. Consequently, epidemiological evidence supports a causal relationship between the use of PPIs and decreased levels of vitamin B12 [47].

Multiple studies have demonstrated the impact of omeprazole on vitamin B12 levels. For example, a trial involving healthy volunteers who underwent a Schilling test before and after two weeks of daily omeprazole therapy revealed a decrease in vitamin B12 absorption. The absorption decreased from 3.2 to 0.9% for those on a 20 mg daily dose, and from 3.4 to 0.4% for those on a 40 mg daily dose [48]. In a case-control study of individuals aged 65 years or older, the prolonged use (> 1 year) of PPIs was connected to an elevated risk of vitamin B12 deficiency [49]. Similarly, a more extensive case-control study involving community-dwelling adults highlighted an increased risk of vitamin B12 deficiency in individuals with prolonged PPI usage [50]. The utilization of omeprazole is additionally linked to decreased average serum levels of Vitamin B12 [51]. Given these findings, it is advisable to periodically assess vitamin B12 levels in individuals on long-term PPI regimens.

#### Intestinal flora disturbance

Gastric acid plays a crucial role in the immune system by effectively eliminating ingested pathogens within the digestive tract. In individuals with normal gastric acid physiology, the gastrointestinal microenvironment becomes unfavorable for the growth of various bacterial and fungal. However, omeprazole use can alkalize the microenvironment, potentially disrupting the gut microbiome and leading to gut microbiota disorders [47, 52].

The intricate interplay between OA and gut microbiota disturbance has emerged as a focal point in recent years [53]. According to the prevailing viewpoint, lipopolysaccharides (LPS) can traverse from the gut into the systemic circulation. They initiate mild inflammation by activating Toll-like receptors on macrophages within the joint cavity, potentially instigating OA [54]. LPS are distinctive constituents of the cell walls of gram-negative bacteria, primarily sourced from gram-negative bacteria in the distal ileum and colon. A prior study revealed a relatively elevated abundance of gram-negative rods in the feces of OA patients [55]. The causal relationship between gut microbiota and the risk of OA has been further validated by a two-sample MR analysis, which identified potentially linking gram-negative bacteria that are causally related to OA [56].

Another perspective suggests that LPS contribute to obesity by inducing metabolic syndrome and insulin resistance [57–61]. For weight-bearing joints like the knees and hips, excess weight inevitably imparts greater stress upon these joints. Macrophages in adipose tissue synthesize pro-inflammatory cytokines such as IL-6 and TNF-α, which might foster a low-grade systemic pro-inflammatory state, thereby triggering OA.

### Clinical implications

PPIs are commonly prescribed for patients with OA, with 44.0% of patients using them [21]. Among them, omeprazole is the most commonly prescribed. Building upon previous cohort studies, this study employed MR analysis to confirm a causal relationship between omeprazole as an exposure factor and the incidence of OA. The findings have significant implications for current clinical practice. Prior research has demonstrated gender and age differences in the prescription patterns of PPIs. Females and the elderly over 60 years old have a higher frequency of taking PPIs [62]. Gender differences in PPI prescription patterns may arise because females are more likely to suffer from symptoms like gastric reflux and dyspepsia, prompting clinicians to prescribe omeprazole more frequently for female patients [63, 64]. Age differences might stem from the greater need for gastric protection through acid-suppressing drugs for patients aged 60 and above who are undergoing NSAIDs or dual antiplatelet therapy [36].

Age is a robust predictor of OA, with incidences of hand, hip, and KOA increasing notably after the age of 50 [65–67]. Female gender is linked to a heightened prevalence and severity of OA. Extensive meta-analyses have shown that females have a higher susceptibility to prevalent and emerging knee and hand OA, as well as incident hip OA, compared to males [68]. Furthermore, females, particularly post-menopause, exhibit pronounced severity of KOA [65, 68]. In light of these factors, we underscore the imperative of granting clinicians due consideration in their prescription practices and engaging in comprehensive evaluations of potential hazards and merits for their patients.

### Strengths and limitations

#### Strengths

Most of the previous studies that report on the link between the use of PPIs and adverse events are observational [47]. Nevertheless, the analysis of such observational data entails a potential for erroneous associations due to factors like confounding and inadequate study design. The validity of connections between omeprazole uses and adverse events is of particular concern, given that omeprazole is frequently prescribed to individuals suffer from multiple diseases simultaneously, and these conditions themselves could be the actual cause of the adverse outcome. This is commonly known as confounding.

In this study, the application of MR effectively mitigated the interference of confounding factors such as social environment and lifestyle, as well as the impact of reverse causation on the outcomes. Sensitivity analysis further enhanced the reliability and stability of the results. The data utilized in the study were derived from a European ancestry population, which showed minimal heterogeneity. Moreover, the GWAS data originated from two independent samples, resulting in a relatively larger sample size, thus maximizing statistical power.

#### Limitations

First, it’s worth noting that the two-sample MR employed in this study only addresses linear associations between exposure and outcome, precluding analyses of non-linear relationships. Secondly, the study only included individuals of European descent, which limits its external validity. As a result, the effects of omeprazole on OA within other ethnic groups remain to be confirmed. Lastly, due to the absence of gender or age-stratified GWAS data, this study could not validate if the association between omeprazole and OA holds homogeneity across different genders or age groups, impeding the further elucidation of its underlying biological mechanisms.

## Conclusion

The study conducted utilizing MR illustrates that omeprazole, as an exposure factor, increases the risk of OA. Clinicians should be vigilant about the prolonged use of omeprazole, especially in populations with already heightened OA risks. However, the effects and underlying mechanisms of omeprazole on OA remain poorly understood. Future research in this field should prioritize long-term, prospective studies to establish the causal relationship between omeprazole use and OA outcomes more definitively. These studies should encompass diverse population groups and investigate variations in omeprazole dosages and treatment durations. Additionally, there is a significant need for mechanistic studies to elucidate how omeprazole influences OA at the molecular and cellular levels, potentially leading to new preventive strategies or alternative treatments. Furthermore, incorporating real-world data to evaluate the impact of omeprazole across various clinical scenarios will provide a more comprehensive understanding of its effects on OA. This approach will be particularly valuable in identifying patient groups more susceptible to OA associated with omeprazole use.

## Data Availability

Corresponding authors can be contacted to request access to the datasets used or analyzed in this study.

## Abbreviations

GWAS: Genome-Wide Association Study
IEU: Integrative epidemiology unit
MR: Mendelian randomization
SNP: Single nucleotide polymorphism
